# A Vision-Based Algorithm for Assessing Head and Hand Tremor: Development and Validation Against IMU Sensors

**DOI:** 10.3390/s26030928

**Published:** 2026-02-01

**Authors:** Slavka Netukova, Jan Tesař, Tereza Hubená, Petr Hollý, Evžen Růžička, Radim Krupička

**Affiliations:** 1 Department of Biomedical Informatics, Faculty of Biomedical Engineering, Czech Technical University in Prague, 272 01 Kladno, Czech Republic; slavka.netukova@fbmi.cvut.cz (S.N.); tereza.hubena@fbmi.cvut.cz (T.H.); 2 Department of Neurology and Centre of Clinical Neuroscience, First Faculty of Medicine, Charles University and General University Hospital, 128 21 Prague, Czech Republic; petr.holly@vfn.cz

**Keywords:** camera, computer vision, contactless measurement, spectral analysis, tremor, video

## Abstract

**Highlights:**

**What are the main findings?**

- Development of a novel center-of-mass algorithm for 2D video tremor analysis.
- Moderate-to-good agreement (ICC 0.70–0.80) with IMU sensors for tremor power.
- Successful validation of hand tremor frequency extraction on 30 participants.

**What are the implications of the main findings?**

- Contactless video analysis serves as a cost-effective alternative to wearable sensors.
- The lightweight algorithm is suitable for integration into telemedicine platforms.
- Open-source implementation enables accessible and remote clinical tremor monitoring.

**Abstract:**

Tremor is the most prevalent human movement disorder, characterized by rhythmic oscillations of a body part. Accurate tremor assessment is essential for diagnosis, monitoring, and treatment evaluation. Traditional methods rely on accelerometry-based measurements, requiring direct sensor attachment, which may be impractical in some settings. We developed a novel algorithm for detecting tremors from video recordings based on the motion of the center of mass and implemented it in the open-source software TremAn3. Motion data were extracted from 2D video recordings of both hands and the head, and spectral analysis was then performed to quantify the tremor by calculating peak tremor power and peak power frequency. A total of 30 videos were recorded from 30 participants with essential or dystonic tremors. Simultaneously, acceleration signals were collected using inertial measurement units (IMUs) placed on the backs of the hands and forehead as a gold-standard reference. Agreement between video- and IMU-derived metrics was assessed using intraclass correlation coefficients (ICCs) and mean absolute error (MAE). For PP, video-based estimates showed moderate-to-good agreement (ICC: 0.70 left hand, 0.77 right hand, 0.80 head) with MAE of 8.12–10.80 dB. For PPF, agreement was moderate for the hands (ICC: 0.60 left, 0.67 right; MAE: 0.54–0.76 Hz) but poor for head PPF (ICC: 0.08; MAE: 2.06 Hz). Our results indicate that video analysis can serve as a viable alternative to traditional accelerometry for tremor quantification. This contactless method holds significant potential for telemedicine and research applications.

## 1. Introduction

A tremor is the most prevalent human movement disorder, characterized by involuntary, rhythmic oscillations of a body part, most commonly the upper limbs. It can occur in various neurological conditions, including essential tremor and Parkinson’s disease, significantly impacting daily activities and quality of life. Accurate quantification of a tremor is crucial for diagnosis, disease monitoring, and treatment effect evaluation. Traditionally, tremor assessment relies on clinical rating scales or sensor-based methods, such as accelerometry and electromyography [1]. However, these approaches often require specialized equipment and in-person assessments. Recent advancements in computer vision and artificial intelligence have enabled the development of video-based analysis of movement disorders [2], offering a contactless, cost-effective alternative with potential applications in telemedicine and large-scale clinical studies.

Hand and head tremors in essential tremor typically occur within a frequency range of 4–12 Hz, whereas dystonic tremor commonly presents at 3–7 Hz, with head tremor often lying at the lower end of this spectrum [3,4]. These frequency characteristics are clinically well established and provide an important reference for interpreting the spectral features extracted in this study.

Wearable sensors have been widely investigated as a way to measure tremors [5,6,7]. Smartphone’s built-in accelerometers have been shown as comparable to laboratory accelerometers in the assessment and classification of tremors [8,9,10,11,12]. Setting up an accelerometry measurement session might be time-consuming and requires special equipment which is strapped to the patient’s hand or needs to be held in the patient’s hand. The latter, contact or interaction of the patient with the equipment, can influence tremor characteristics.

Six previous studies focus on tremor analysis via video recordings. Soran et al. [13] performed skin detection based on pre-trained skin color to detect a hand in the video sequence followed by the detection of motion via an optical flow method. Williams et al. [14] and Hemm-Ode et al. [15] applied an optical flow algorithm to videos of two patients with a hand tremor. However, the authors of these studies did not report any quantitative measurements of tremor frequency. Uhríková et al. [16] detected a tremor from a video by looking at changes of the red color intensity in time. Pintea et al. [17] employed Lagrangian and Eulerian approaches to detect tremors. The latest study published by Williams et al. [18] determined tremors via optical flow from a smartphone video. In addition to these studies, Alper et al. [19] proposed a hybrid method combining hand motion tracking and optical flow to improve tremor quantification accuracy. This approach further demonstrates the feasibility of extracting tremor characteristics from conventional 2D video recordings, supporting the development of contactless and clinically applicable tools.

In order to keep tremor research available and potentially transferable to clinical practice, the algorithms for video processing should be simple, efficient, not computationally intensive, and accurate. Such an algorithm has the potential to be switched from research development environments (e.g., Matlab) to other platforms with practical applications (e.g., smartphone, desktop, cloud application).

The aim of this study is to introduce and validate a simple, transparent, and computationally efficient video-based method for tremor quantification. The main contributions are as follows: (i) we present a lightweight center-of-mass tracking approach that enables tremor estimation from standard 2D video without relying on complex optical-flow or deep-learning frameworks; (ii) we extract clinically relevant tremor characteristics using spectral analysis of the motion signals from the hands and head; and (iii) we perform a systematic validation of these video-derived metrics against simultaneously recorded IMU measurements. Together, these contributions highlight the feasibility of a practical and accessible contactless alternative to sensor-based tremor assessment in both research and clinical settings.

## 2. Materials and Methods

### 2.1. Participants

Neurological patients with visible hand and/or head tremors were recruited from the Movement Disorder Center of the Department of Neurology, General University Hospital in Prague. In total, 30 patients (22 women, 8 men, mean age 64.1 ± 13.3 years) were included. The present sample includes patients with essential tremor or dystonic head tremor who were recruited as part of earlier studies [20,21]; the current analysis focuses on synchronized accelerometric and video recordings obtained during the same assessment sessions. The sample size was selected for an initial proof-of-concept validation of the proposed video-based algorithm against IMU-derived reference metrics. The research protocol was approved by the local research Ethics Committee of the General University Hospital, Prague, according to the Declaration of Helsinki and an informed consent was obtained from all participants before entering the study.

### 2.2. Procedure

A video camera Logitech BRIO 4K PROWEBCAM (Logitech International S.A., Lausanne, Switzerland) was placed on a tripod so that the upper body and head of the patient could be comfortably captured. The distance from camera to patient was not tightly controlled, but in practice it was around 2 m. All recordings were performed in the same examination room under stable lighting conditions. Natural daylight was used whenever possible, and artificial fluorescent lamps were switched off during most sessions to avoid flicker artifacts. Only six recordings were affected by artificial light, which produced visible flickering in the video signal. These cases were identified and handled as described in the Spectral analysis subsection. Data was recorded at 30 frames per second and 1280 × 720 pixel resolution. Each participant contributed one recording (*N* = 30), resulting in 30 video files of 20–22 s each (≈600–660 frames per video; total ≈ 10–11 min). Videos were stored as MP4 (H.264/AVC).

Three-axial inertial measurement units (MTw Awinda Wireless 3DOF Motion Tracker; Movella Inc., Enschede, The Netherlands) were attached to the dorsum of each participant’s hands using Xsens gloves and attached to the head using adjustable straps provided by the manufacturer. Acceleration was recorded contemporaneously with the video (without an exact time-lock mechanism) at a sample rate of 100 Hz. IMU data were exported in CSV at 100 Hz, yielding ≈ 2000–2200 samples per axis per recording. Overall, the dataset consists of 30 paired acquisitions (video + IMU). In this study, no post hoc synchronization between IMU and video was performed. The analyses were limited to frequency-domain metrics (peak power and peak power frequency) computed independently for each modality over the same nominal recording interval (20–22 s), after trimming any obvious transients. Because these metrics are invariant to relative time shifts under steady-state conditions, explicit sample-level alignment was not required.

**Figure 1:**
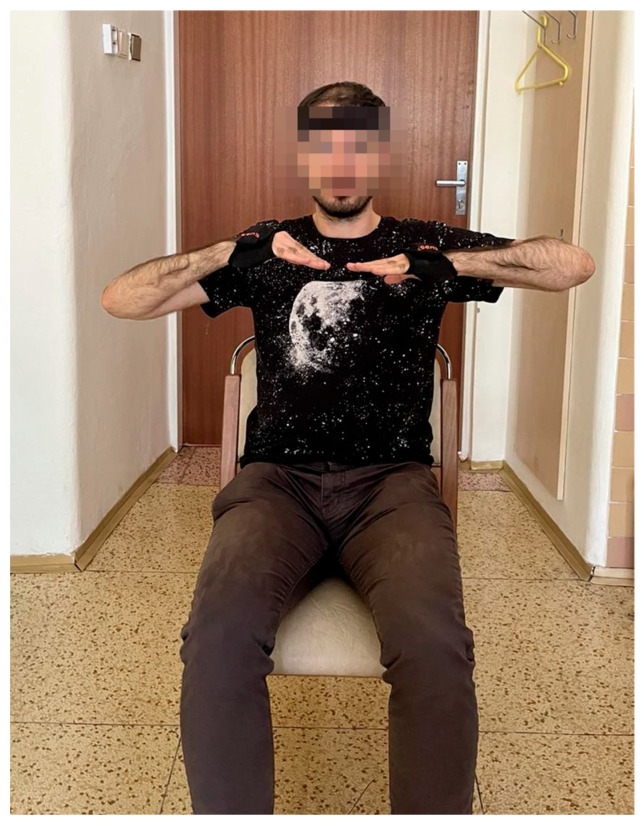
Example of participant measurement while performing the wing arm position (SWing) test with accelerometers attached to the dorsum of both hands and the forehead.

The participants were seated and filmed from the frontal view while in the wing arm position (SWing), Figure 1. They were instructed to assume the SWing pose before the start of each recording, and acquisition began only after the examiner verified that the participant was stable in the required position. In occasional cases where the recording started slightly earlier, the initial segment containing voluntary movement was trimmed during preprocessing to avoid motion artifacts. The recording setup was standardized to minimize visual noise and background motion. All videos were captured against a plain, non-textured background with the camera fixed on a tripod to ensure frame-to-frame stability. No other moving objects were present within the camera’s field of view. Each recording was between 20 and 22 s long.

### 2.3. Data Analysis

Data was imported and processed using MATLAB (version R2019b; MathWorks Inc., Natick, MA, USA). After a visual inspection of the video recordings, both recordings (video as well as acceleration) were properly trimmed if needed (e.g., to avoid side effects from voluntary movement artifacts as the patient settled into position). The videos and acceleration signals were processed with a custom-written MATLAB code.

### 2.4. Video Processing

First, regions of interest (RoI) were manually drawn around the hands for one frame of each video. The RoI is defined as a rectangular area and is selected by mouse dragging. Separate RoIs were defined for the right and left hands in the first frame of each video. The typical RoI size was approximately 150 × 120 pixels, depending on the participant’s hand size and position in the frame. In addition to the hand RoIs, a separate RoI was defined for the head in each video. This rectangular RoI encompassed the forehead and whole facial area and was selected at the start of the task. Each RoI was defined as slightly larger than the hand or head to ensure that it remained fully within the selected region throughout the entire recording. No cases were observed in which the body part moved outside the RoI. It needs to be selected only once for the entire video.

**Figure 2:**
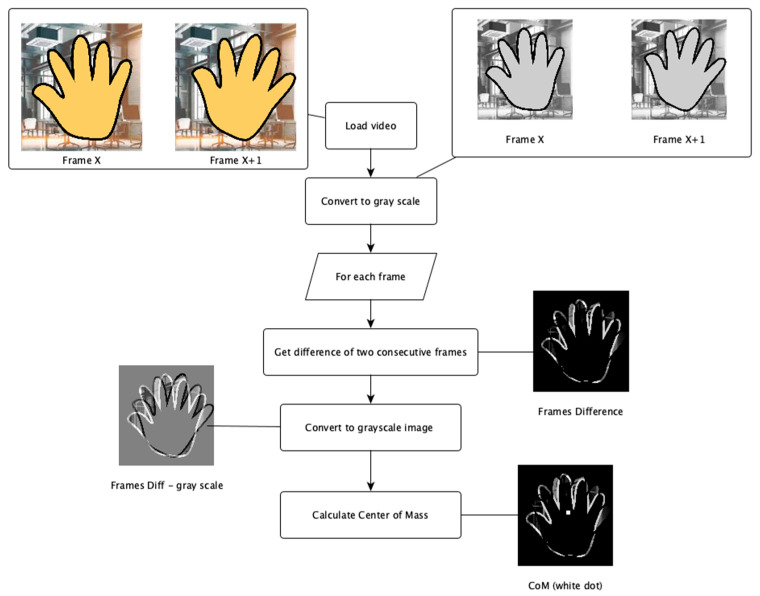
Algorithm of center of mass computation.

Second, the time series of the Center of Mass (CoM) was calculated for each Region of Interest (RoI). The processing pipeline included converting the RoI to grayscale and calculating the difference between two consecutive frames. Frame differences were normalized to a range of 0 to 1 to ensure consistency across different intensity levels. The CoM was calculated in the way, where pixel intensities are treated as masses, and the coordinates $\left(x_{c},\;y_{c}\right)$ are determined using weighted averages:

(1) $$x_{c}=\frac{\Sigma x\cdot I(x,\;y)}{\Sigma I(x,\;y)},$$

(2) $$y_{c}=\frac{\Sigma y\cdot I(x,\;y)}{\Sigma I(x,\;y)}$$

where I(x, y) is the intensity at pixel $\left(x,\;y\right)$ coordinates. This approach provided a time series of two CoM coordinates (x and y) for each frame. The process is depicted in Figure 2. RoIs were saved as pixel coordinates (x, y, width, height) in CSV to enable reproducible re-processing.

Finally, a single time series representing RoI motion was obtained so that the L1 norm (defined as the sum of the absolute values of a vector’s components) was applied to CoM coordinates. The full implementation of the video-processing algorithm is openly available at: https://github.com/tesar-tech/treman_algorithms (accessed on 27 January 2026).

### 2.5. Spectral Analysis

The power spectral density (PSD) was computed using the Welch method with a Hamming window and 50% overlap. The sampling frequency was set to 30 Hz. Signal durations varied between 20 and 22 s. The Welch method automatically adjusted the window length based on signal duration, resulting in a frequency resolution of approximately 0.4 Hz for 20-s recordings and 0.366 Hz for 22-s recordings. Given the minimal difference between these values (0.034 Hz), the frequency resolution was considered effectively consistent across all recordings. Thus, three PSDs were obtained for both the acceleration signals (from the left hand, right hand, and head IMUs) and the corresponding CoM position signals derived from the three ROIs (left hand, right hand, and head). The L1 norm was applied to combine individual axes into one. Then, we removed frequencies below 2 Hz (low-frequency drifting movements) and above 12 Hz (higher than hand tremor).

The following features were computed from power spectral analysis: peak power (dB) and peak power frequency (Hz). The peak tremor power (PP) was defined as the maximum amplitude of the PSD within the 2–12 Hz frequency range, representing the strongest oscillatory component of the movement. The peak power frequency (PPF) was defined as the frequency at which this maximum power occurred, corresponding to the dominant tremor frequency. Both parameters were computed from the L1-norm of the PSD obtained from the x- and y-coordinates of the center-of-mass (CoM) time series.

During six measurement sessions, when artificial lighting in the room was turned on, the video recordings exhibited pronounced power peaks near the cut-off frequencies (i.e., 2–4 Hz and 10–12 Hz). These false peaks were caused by the flickering of fluorescent lamps, combined with the properties of CMOS sensors, which induced the “rolling shutter effect”. These peaks were easily identifiable by their narrow bandwidth and simultaneous presence across all ROIs (hands and head). Importantly, the 2–4 Hz and 10–12 Hz ranges were not globally excluded from the analysis. The indicated thresholds were used only to recognize and disregard artificial lighting artifacts in the affected recordings. True tremor components within the physiological range (typically 3–7 Hz for dystonic tremor and 4–12 Hz for essential tremor) were always retained.

### 2.6. Statistical Analysis

Statistical analysis was performed with MATLAB (version R2019b; MathWorks Inc., Natick, MA, USA). The peak power and peak power frequency derived from each video was compared with their counterparts derived from the accelerometer. The level of concordance was quantified by the intraclass correlation coefficient (ICC). The selection of a proper definition was done according to guidance from Koo and Li [22]. In this investigation, we used a Two-Way Random Effects Model, Single Measures, Consistency with the confidence intervals derived in Fleiss and Shrout [23]. ICC values less than 0.50, between 0.50 and 0.75, between 0.75 and 0.90, and greater than 0.90 indicated poor, moderate, good, and excellent reliability, respectively [24]. Next, we calculated the mean absolute error (MAE) for both tremor metrics. The concordance of measurement systems is visualized with Bland–Altman plots.

## 3. Results

**Figure 3:**
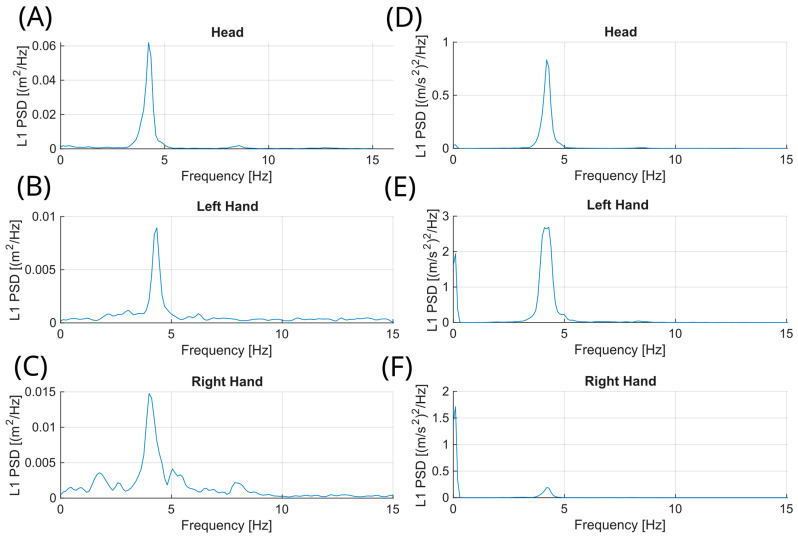
L1-norm motion signals computed from (**A**–**C**) CoM displacement in the video ROI and from (**D**–**F**) acceleration signals recorded by the IMUs. Shown are signals for the (**A**,**D**) head, (**B**,**E**) left hand, and (**C**,**F**) right hand.

**Figure 4:**
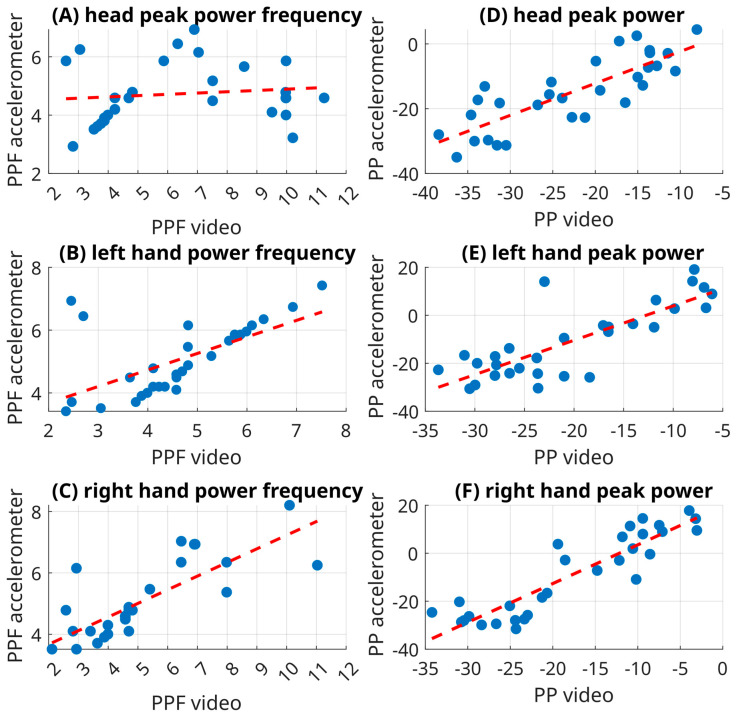
Correlation plots comparing peak power (PP) (**A**–**C**) and peak power frequency (PPF) (**D**–**F**) derived from video-based CoM analysis and IMU accelerometry for the head (**A**,**D**) and both hands (left (**B**,**E**); right (**C**,**F**)). Each panel shows the linear regression fit (dashed red line) and illustrates the degree of agreement between modalities. The hand measurements demonstrate moderate to good correlation for both PP and PPF, whereas the head exhibits weaker agreement, particularly for PPF, reflecting the different biomechanical characteristics of rotational head tremor and the limitations of 2D video analysis for capturing such motion.

**Figure 5:**
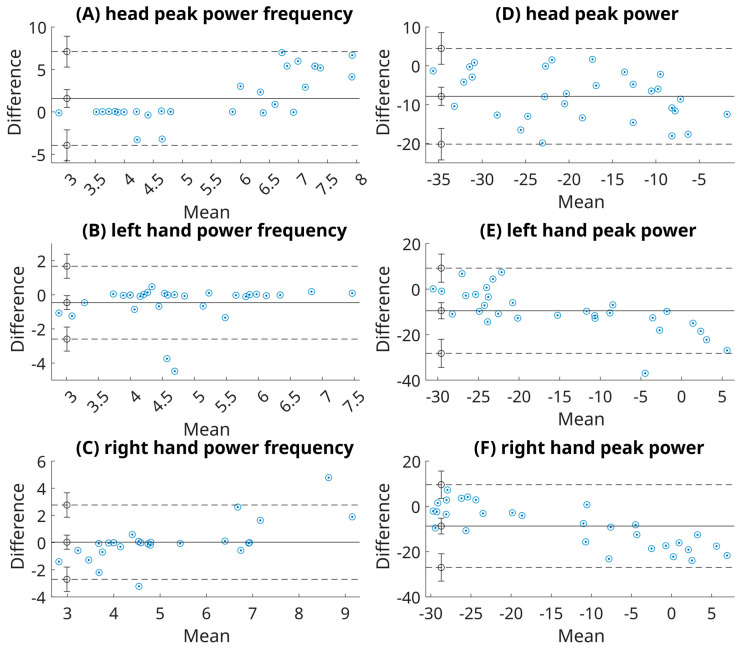
Bland–Altman plots illustrating the agreement between video-derived and IMU-derived tremor metrics for peak power (PP) (**A**–**C**) and peak power frequency (PPF) (**D**–**F**) for the head and both hands. The plots show the mean difference (solid line) and 95% limits of agreement (dashed lines). The results indicate relatively tight agreement for the hand measurements (left (**B**,**E**); right (**C**,**F**)), whereas the head exhibits (**A**,**D**) wider limits of agreement and greater variability, again reflecting the differences in how rotational head motion is captured by the two modalities.

Out of six cases, the ICC values of two cases exceeded 0.75, indicating good reliability. Namely, the peak power of the right hand and head showed an ICC of 0.70 and 0.80, respectively. Three cases (both tremor metrics derived for the left hand and peak power frequency of the right hand) demonstrated moderate reliability (0.70 for peak power of the left hand, 0.60 for peak power frequency of the left hand, and 0.67 for peak power frequency of the right hand). The head peak power frequency was poor (rho = 0.08), suggesting that head PPF was unreliable in our data, potentially due to the biomechanical characteristics of cephalic tremor. For details, refer to Table 1 and Table 2. The tremor parameters in question for all participants are depicted in Figure 3, Figure 4 and Figure 5.

## 4. Discussion

In the present study, the capability of an ordinary 2D video camera to capture tremor features using a novel video processing algorithm was evaluated by investigating the intra-class correlation between the tremor features derived from video recording and from acceleration sensors. We found that the results of our video processing algorithm showed strong agreement with accelerometer-derived measurements under the tested conditions. It should be noted that the validated range of frequencies was limited by the pathology of the patients in the tested group, typically within a frequency range of 4–12 Hz for ET and 3–7 Hz in dystonic tremor [3,25]. Accordingly, because our primary aim was methodological validation rather than exhaustive tremor phenotyping, we focused on this expected range; atypical components outside it were not systematically assessed. This focus may limit the generalizability of our findings to tremor phenotypes with lower dominant frequencies (e.g., Parkinsonian tremor), which were not represented in our cohort.

The observed difference in agreement for head tremor does not indicate a failure of the method but rather highlights the fundamentally different motion characteristics these modalities capture. Head tremor is predominantly rotational, occurring around three anatomical axes: yaw (no-no), pitch (yes-yes), and roll (lateral tilt). Gyroscopic sensors are specifically designed to measure such angular velocity directly. In contrast, accelerometers detect translational acceleration, which represents rotational motion only indirectly and with accuracy that depends on the sensor’s distance from the axis of rotation [26]. For instance, yaw or pitch head tremor may result in horizontal or vertical displacement of an accelerometer, respectively, but the signal is attenuated if the sensor is near the axis of rotation. Similarly, roll (rotation about the *z*-axis) may be poorly captured by both accelerometers and standard video analysis, depending on sensor or camera positioning. While 2D video analysis can detect visible displacement—particularly in the image plane—it lacks the depth sensitivity and angular specificity of gyroscopic sensors. Markerless 3D video systems (e.g., Microsoft Kinect) may theoretically capture such motion, but their spatial and temporal resolution is insufficient for reliably detecting small-amplitude tremor [19]. Consequently, these modality-specific limitations in capturing rotational head motion account for the higher MAE observed for the head compared with the hands. This insight is important for selecting the appropriate measurement tool for a given clinical or research question.

Despite these methodological distinctions, video analysis offers significant practical advantages. It provides a contactless method for tremor evaluation, which is beneficial in clinical scenarios where sensor placement is impractical or could interfere with the natural expression of tremor. While modern wireless IMUs are highly practical and accurate, video recording can be performed using readily available devices, such as smartphones, making it a highly accessible and low-cost assessment tool. The presented algorithm also allows for feedback on tremor frequency and power and supports simultaneous analysis of multiple regions of interest (RoIs), including coherence estimation between them. To our knowledge, such functionality is not currently available in standard IMU-based systems. A step towards greater clinical applicability would be to implement the present algorithm as a desktop or mobile application for tremor analysis. One distinct advantage of video-based methods is the ability to retrospectively analyze archived video segments, which may be valuable for post hoc syndromological assessment or for tracking tremor progression over time when tremor frequency, power, or coherence are of interest. provided that the video meets the minimum sampling requirements (i.e., a sampling rate of at least twice the maximum expected tremor frequency, per the Nyquist theorem). It should be noted that the analytical value of legacy videos is highly dependent on recording conditions such as camera stability, distance, and lighting. Without these being well-controlled, the quality and reliability of tremor analysis may be severely compromised.

Another practical consideration concerns lighting conditions during video acquisition. While the majority of sessions were performed under stable daylight, a few recordings were affected by fluorescent flicker. Because the algorithm is based on inter-frame intensity differences rather than absolute brightness, moderate illumination changes had negligible influence on frequency-domain outcomes. Nonetheless, variable lighting remains a common challenge for vision-based motion analysis, and future developments will include adaptive illumination normalization to further improve robustness.

Next, the presence of background motion or visual clutter is another factor that may influence the reliability of frame-difference-based analysis. Although our recordings were standardized to minimize such effects, the method remains inherently sensitive to complex backgrounds. Future versions of the algorithm will incorporate background subtraction or adaptive spatial filtering to further reduce noise interference.

As a technical proof-of-concept, this study was intentionally focused on tremor frequency, which does not in itself provide a complete clinical picture. Accordingly, our primary aim was to demonstrate a reliable, contactless approach for frequency measurement.

A key consideration for the current implementation is its reliance on manual RoI definition. This introduces potential variability and may be challenging in tasks with large-range movements. Our analysis also revealed that the algorithm’s performance is dependent on the visual characteristics of the selected RoI; low-contrast or poorly textured areas may yield less reliable results. This finding highlights the importance of consistent ROI selection, with larger, higher-contrast regions providing a more stable signal. Although the manual definition of the RoI introduces a small degree of subjectivity, it was deliberately used in this validation study to maintain full control over the analyzed regions and to ensure spatial consistency across recordings. The RoI selection required only a single click-and-drag action per video, typically taking less than five seconds. This manual step therefore represents a negligible practical burden while guaranteeing consistent reference areas for comparison. To address this, future work will explore automated RoI tracking and motion-adaptive segmentation. We note that intra- and inter-operator repeatability of the manual RoI placement was not evaluated in this study; a dedicated repeatability assessment with repeated RoI placement by multiple raters is planned.

The absence of hardware or software synchronization between the IMU sensors and the video stream poses a minor limitation of the present study. In particular, the lack of precise temporal alignment prevents any evaluation of time-domain correspondence or temporal/phase consistency between modalities (e.g., coherence or other time-resolved comparisons). However, because our analyses focused exclusively on frequency-domain parameters (peak power and peak power frequency), which are largely insensitive to small temporal shifts, the lack of synchronization is unlikely to meaningfully affect the results. While this setup does not allow phase- or coherence-based analyses, it still supports a robust comparison of summary spectral metrics across modalities, and any potential bias in absolute power comparisons between modalities is expected to be minimal.

Another characteristic of this uncalibrated approach is that the derived tremor power is a relative measure, influenced by camera distance and angle. While these values are suitable for within-subject comparisons under consistent conditions, they do not represent absolute tremor amplitude. However, frequency analysis alone is clinically valuable, particularly for distinguishing tremor types, such as functional versus organic tremors [27]. Future research may also explore potential strategies for amplitude calibration.

Based on a recently published overview [4], the present algorithm demonstrates considerable potential for telemedicine applications, particularly for remote or home-based tremor assessment. A promising direction for future development is the implementation of real-time video processing and its integration into clinical diagnostic and monitoring tools, which would further enhance the method’s practicality and accessibility.

Finally, we identified several environmental and analytical factors to consider. Recordings under some artificial lighting conditions introduced flicker artifacts. Based on this, we recommend recording under natural lighting conditions whenever possible, and we plan to develop adaptive filtering techniques to mitigate such artifacts. In the current analysis, spectral peaks were identified heuristically. To enhance objectivity, future iterations will incorporate algorithmic peak detection approaches, such as prominence-based thresholding. The scope of this validation was focused on the head and hands; therefore, future research should extend the validation to other body parts and tremor types, and determine the minimum detectable tremor amplitude. A final step towards greater clinical applicability will be to implement the present algorithm as a user-friendly desktop or mobile application.

The key innovation of the proposed algorithm lies in its simplicity and transparency. By relying on the motion of the center of mass rather than complex deep-learning or optical-flow frameworks, our method achieves efficient tremor quantification with minimal computational cost and without the need for large training datasets. This design makes the approach highly adaptable for both research and clinical use, particularly in low-resource or remote environments. Moreover, the open-source implementation facilitates reproducibility and further development by the research community.

In contrast to optical-flow-based approaches, which estimate dense pixel-wise motion fields to capture local displacements, our method quantifies the global movement of a selected region of interest through the computation of its center of mass (CoM). This simplified motion descriptor reduces computational complexity and sensitivity to lighting or texture variations while maintaining sufficient temporal precision for spectral tremor analysis. Although optical-flow methods can provide more spatial detail, their higher computational cost and dependence on visual conditions limit their practicality in clinical and telemedicine contexts. The proposed CoM approach therefore represents a robust and accessible alternative for contactless tremor assessment.

## Figures and Tables

**Table 1:** Intraclass correlation coefficients (ICCs), mean absolute error (MAE; dB), relative MAE (%), and descriptive statistics (mean ± SD) of peak power for video-derived (PP video; dB) and IMU-derived (PP IMU; dB) measurements for all three regions of interest.

		ICC				PP IMU	PP Video
	rho	Lower Limit	Upper Limit	MAE [dB]	Relative MAE [%]	Mean (SD) [dB]	Mean (SD) [dB]
Left Hand	0.70	0.45	0.84	10.80	101.31	−20.17 (8.53)	−10.66 (15.06)
Right Hand	0.77	0.57	0.88	10.23	112.57	−17.79 (9.52)	−9.09 (17.00)
Head	0.80	0.63	0.90	8.12	54.46	−22.76 (9.07)	−14.91 (10.92)

**Table 2:** Intraclass correlation coefficients (ICCs), mean absolute error (MAE; dB), relative MAE (%), and descriptive statistics (mean ± SD) of peak power frequency for video-derived (PPF video; dB) and IMU-derived (PPF IMU; dB) measurements for all three regions of interest.

		ICC				PPF IMU	PPF Video
	rho	Lower Limit	Upper Limit	MAE [dB]	Relative MAE [%]	Mean (SD) [dB]	Mean (SD) [dB]
Left Hand	0.60	0.31	0.79	0.54	10.71	4.58 (1.31)	5.04 (1.13)
Right Hand	0.67	0.41	0.83	0.76	15.06	5.07 (2.11)	5.04 (1.2)
Head	0.08	−0.28	0.42	2.06	43.56	6.31 (2.75)	4.73 (1.01)

## Data Availability

The data presented in this study are available on request from the corresponding author. The data are not publicly available due to privacy and ethical restrictions, as they contain sensitive personal data of patients, including video recordings.
